# Treatment with direct oral anticoagulants in patients with upper extremity deep vein thrombosis

**DOI:** 10.1186/s12959-017-0149-x

**Published:** 2017-10-03

**Authors:** Francisco Sánchez Montiel, Raein Ghazvinian, Anders Gottsäter, Johan Elf

**Affiliations:** Department of Vascular Diseases, Lund University, Skåne University Hospital, S-205 02 Malmö, Sweden

**Keywords:** Venous thromboembolism, Upper extremity deep vein thrombosis, Direct acting oral anti coagulants

## Abstract

**Background:**

Upper extremity deep vein thrombosis (UEDVT) constitutes around 10% of all DVT, and can cause both pulmonary embolism (PE) and postthrombotic syndrome (PTS) in the arm. The incidence of secondary UEDVT is increasing due to widespread use of central venous catheters in patients with cancer and other chronic diseases. The safety and efficacy of using new direct acting oral anti coagulants (DOAC) in the treatment of UEDVT has not been systematically evaluated. Our aims were to evaulate efficacy, safety, and risk of recurrence of venous thromboembolism (VTE) during DOAC treatment in UEDVT patients.

**Methods:**

Data from the Swedish national anticoagulation registry (AuriculA) was retrospectively evaluated for all 55 patients (27 men aged 23–86 years, and 28 women aged 18–75 years) treated with DOAC because of UEDVT between 2012 and 2015 in the southernmost hospital region of Sweden with 1.3 million inhabitants in 2016. Patients were followed for 6 months.

**Results:**

During 6 months after institution of DOAC treatment there was one recurrence (2%) of DVT during treatment and two (4%) recurrences after cessation of treatment. No patient died, whereas one (2%) suffered a clinically relevant nonmajor bleeding.

**Conclusion:**

DOAC can be used in the treatment of UEDVT patients with acceptable efficacy and safety.

## Background

Venous thromboembolism (VTE), including deep vein thrombosis (DVT) and pulmonary embolism (PE) affects ≈5% of the population during life [1]. VTE is treated with anticoagulant (AC) therapy for a minimun of 3 months to prevent thrombus extension or embolization, recurrences, and posttrombotic syndromes (PTS) [2]. Hereafter, the decision to stop or continue treatment depends on the balance between the risk of recurrence (1–10%/year) and bleeding (2–4%/year) [1].

Upper extremity DVT (UEDVT) constitutes around 10% of all DVT, and can cause both PE and PTS in the arm [3, 4]. The incidence of secondary UEDVT is increasing due to widespread use of central venous catheters (CVC) in patients with cancer and other chronic diseases [3, 4].

During recent years novel direct acting oral anticoagulants (DOAC) with a favorable risk profile have been increasingly used as an alternative to warfarin for VTE treament [5, 6]. Furthermore, the need for monintoring of DOAC treatment is less than for warfarin, simplifying outpatient treatment of VTE [6]. Whereas DOAC are nowadays considered as first alternative for treatment of PE and DVT in the lower extremities [7], the safety and efficacy of using DOAC for treatment of UEDVT has not been systematically evaluated.

The Swedish quality registry for patients treated with AC, AuriculA [8], includes data on patient characteristics, treatment, and complications for patients on different kinds of oral AC. AuriculA currently holds data on >25,000 patients from the southernmost hospital region in Sweden, about 15–20% of whom currently use DOAC, the indication being VTE in 15–20%.

Real life data are valuable for clinical decisions upon AC treatment. AuriculA offers ample opportunities for collection of such data, which can be related to clinical outcomes obtained through prospective follow up of registries and patient files. We therefore used AuriculA data to clarify risks for VTE recurrence, death, and bleeding during 6 months in 55 consecutively registered patients with UEDVT treated with DOAC.

## Methods

### Patients

We extracted data from AuriculA [8] for all 55 patients in the southernmost hospital region in Sweden (1.3 million inhabitants) that had been treated with DOAC for UEDVT between 2012, when DOAC were first introduced in Sweden, and 2015.

The following risk factors for UEDVT were recorded and reviewed in digital medical charts and imaging databases: malignancies diagnosed prior to or at diagnosis of UEDVT, peripheral vein catheter (PVC) inserted <1 week of diagnosis, use of CVC, cardiac pacemaker or oral contraceptives (OC), pregnancy or postpartum period (defined as the first 6 weeks after delivery), family history of VTE defined as a history of VTE in first-degree relatives and thrombophilia work up (except analysis of lupus anticoagulant due to the risk of false positive results during treatment with DOAC), immobilization defined as ≥3 days of bedrest, trauma to the upper limb or major surgery within the previous 3 months and reported recent strenuous activity of the affected arm (effort thrombosis). Data on symptomatic recurrent VTE (all locations), mortality, bleeding complications and a diagnosis of post thrombotic syndrome (PTS), during 180 days (6 months) after diagnosis were obtained from review of hospital records and imaging databases.

### Statistical analysis

Descriptive statistics were used to outline patient characteristics. Results are expressed as mean ± SD or n (%). Excel® 2013 (Microsoft® Office Professional Plus) was used for all analyses.

## Results

Baseline data and are available in Table 1. Symptoms reported by the 55 patients were either acute discomfort or swelling of the arm. At least one injection of low molecular weight heparin (LMWH) had initially been given to 38 (69%) patients, whereafter DOAC therapy had been prescribed for 3–6 months or indefinitely. The duration of treatment was 3–6 months if the patient had a UEDVT provoked by transient factors, and indefinite in patients with recurrent unprovoked VTE or strong permanent risk factors. The majority (37[67%]) of patients were treated in hospital (admitted to the hospital >24 h), whereas 18(33%) were treated at an outpatient basis (discharged directly from the emergency unit or admitted to the hospital <24 h). The vast majority (46[84%]) were treated with rivaroxaban, whereas 7(13%) and 2(4%) got apixaban and dabigatran, respectively. Fourty-four (80%) patients started DOAC treatment within 7 days of diagnosis.

**Table 1:** Baseline characteristics in 55 patients with upper extremity deep vein thrombosis (UEDVT) treated with direct oral anticoagulants (DOAC)

Average age (SD)	49 ± 16,9
Male/Female gender	27/ 28
Ongoing tobacco use	26(47)
Malignancy	10(18)
Surgery within 3 months	13(24)
Immobilisation within 3 months	8(15)
Trauma within 3 months	10(18)
Ongoing pregnancy or postpartum period	0(0)
Oral contraception (females)	7(25)
Acute concomitant medical illness	4(7)
Inpatient treatment for UEDVT	37(67)
Central venous catheter	4(7)
Cardiac pacemaker	1(2)
Peripheral venous catheter	15(27)
Trombophilia (among tested)	15(44)
Effort trombosis	3(5)

N(%)

During 6 months, one (2%) 45 year old male patient experienced recurrence of UEDVT. He was heterozygous for factor V Leiden, had BeÇhet’s disease, and had previously been treated with endovascular coiling of a small cerebral aneurysm, His index UEDVT was diagnosed shortly after removal of a CVC from the left jugular vein. After 3 months of rivaroxaban (15 mg BID for 3 weeks followed by 20 mg OD) treatment he once again experienced that his left arm and was more swollen and he also had signs of superior vena caval syndrome. CT-scan showed thrombus in the left brachiocephalic vein and superior vena cava. Treatment was then switched to LMWH bridged to permanent warfarin treatment.

During the 6 months of follow-up, but after cessation of DOAC treatment, two other young male patients (4%) developed recurrent episodes of ipsilateral UEDVT. One was a current smoker and was homozygous for factor V Leiden, the other patient had a positive family history of VTE but was considered to have a primary UEDVT.

No patient died during 6 months of follow-up, whereas one patient (2%) suffered an epistaxis in need for medical attention, classified as a clinically relevant nonmajor bleeding (Table 2).

**Table 2:** Six months follow-up of 55 patients with upper extremity deep vein thrombosis (UEDVT) treated with direct oral anticoagulants (DOAC), N(%)

Death	0(0)
DVT recurrence on treatment	1(2)
DVT recurrence after cessation	2(4)
Major bleeding	0(0)
Clinically relevant nonmajor bleeding	1(2)
Treatment change	1(2)
Post thrombotic syndrome	6(11)
Prescribed permanent anticoagulation at 6 months	6(11)

The type of DOAC treatment was changed only in one (2%) female patient, from rivaroxaban to dabigatran due to a suspected allergic reaction. After 6 months of follow-up, 6(11%) patients had been prescribed permanent DOAC treatment because of estimated high risk of VTE recurrence.

## Discussion

DOAC are shown to be both effective [9–14] and safe [9–14] for VTE treatment, and are therefore recommended as first treatment option [7] both in patients treated for DVT in the lower limb and in patients with PE. Furthermore, our study confirms that DOAC are instituted in clinical practise also in many UEDVT patients, despite the lack of evidence from randomized studies and specific recommendations for this patient group. In this context, it should be noted that we studied patients treated with DOAC already from 2012, when these compounds first became available in our country for VTE treatment. Physicians have herafter become more experienced with the use of DOAC for VTE treatment; the proportion of DVT patients receiving DOAC has increased substantially and is now around 25% in a large European registry based material [15]. The same increasing use of DOAC treatment has presumably also occurred among UEDVT patients. As no systematic evaluation has been done in patients with UEDVT on DOAC treatment, however, the increasing popularity of DOAC made it even more important to evaluate the outcome for 55 patients treated with DOAC for UEDVT and consecutively registered in our quality registry.

We found that only one patient (2%) had recurrence of DVT during treatment, a recurrence rate comparable with randomized controlled trials for the treatment of DVT in the lower limb, as summarized in meta-analysis [16]. The two VTE recurrences occurring after cessation of DOAC therapy cannot be attributed to therapy failure, but instead reflect the innate risk of recurrence that these patients have, although this risk is considered lower compared to patients with DVT in the lower extremity [3]. Furthermore, no major bleedings occurred and only one patient experienced a nonmajor bleeding, figures comparing favorably with bleeding rates in previous large randomized study materials [16], from which patients perceived as having increasing bleeding risk are often excluded.

It is important to note that patients with UEDVT differ from those with DVT in the lower limb and PE, as they more often have secondary thromboses caused by for example CVC and cancer [3, 4]. The recommended treatment in patients with VTE and cancer is LMWH [1, 7], and oral anticoagulation is therefore avoided in a large proportion of UEDVT patients. The 10 patients with a diagnosis of cancer in our study either had strong personal objections to daily LMWH injections or warfarin therapy, or were without active ongoing cancer therapy.

It is also important to note that 20 of our 55 patients had either a PVC, a CVC, or a cardiac pacemaker with intravenous leads inserted in the affected region, and that this implantation had often occurred shortly before the diagnosis of UEDVT. This indicates that our patients have a fairly high rate of concomitant illness, which might also have excluded many of them from entry into randomized studies of VTE treatment. The concept of DOAC treatment of cancer-associated VTE is currently under debate [17, 18], but when rivaroxaban was recently evaluated in 70 cancer patients with UEDVT related to CVC, 13% experienced bleeding complications and one case of fatal pulmonary embolism occurred [19].

Due to logistic reasons, objective verification of a VTE diagnosis with imaging is often postponed to office hours. As in the randomized studies [9–13], many of our patient therefore recieved at least one injection of LMH before imaging and establishment of diagnosis. Hereafter, it is important to note that one third of our patients were treated as outpatients, however, enabling potential savings in addition to the gains offered by DOAC due to the lower need for surveillance in comparison to warfarin [20]. Assessment of the number of days in hospital is essential when evaluating true cost-benefit of the new anticoagulants.

Our study has several limitations, the most important is the fact that is a retrospective clinical follow-up and not a randomized prospective study with a relevant control group. A comparison with UEDVT patients treated with warfarin or LMWH would have suffered from severe inclusion bias, however; warfarin treated patients would probably more often have renal failure, and LMWH treated patients more often cancer or ongoing pregnancy. Furthermore, even if we studied all UEDVT patients treated with DOAC in an area with 1.3 million inhabitants, the material is small in comparison to international registry data [15]. Nevertheless, our data enable the below conclusion.

## Conclusion

DOAC can be used for treatment of patients with UEDVT with acceptable recurrence and bleeding rates.
